# Editorial Department

**Published:** 1857-01

**Authors:** 


Prof. Hamilton has been kind enough to hand us the following interresting letters for publication:

BELFAST, Oct. 18, 1856.

*Dear Doctor:*

The October number of the Buffalo Medical Journal, has this evening arrived freighted with much useful information.

The "cases of fractures" of the spine, reported by yourself, I have perused with a high degree of interest, the more, perhaps, from an accident having occurred the present week in the village of Oramel, producing death in twenty hours, which I shall take the liberty of reporting to you.

Oct. 15th, Mr. McKenna, of Cuba, aged about 40 years, was riding in a lumber wagon, seated upon a bag of oats, which was placed upon a barrel, the barrel rolled and he lost his balance, falling upon the ground, and striking on the back of his head and neck.

Dr. Burr, of Oramel, saw him immediately; he was perfectly conscious, and could speak distinctly; but his legs and arms were paralyzed.

I saw him with Dr. Burr, six hours after he had received the injury. His position and appearance did not indicate any serious difficulty. He talked quite easily, giving me a full account of the manner he was hurt, his present feelings, age, name, &c. His inspiration was natural, but his expiration was rather quick; pulse 68, and full; the temperature of the whole body was slightly elevated; he was nauseated at times; he complained of pain in the back part of the neck; his legs and arms were paralyzed. There was no sensation in front below the nipples, while upon his back sensation continued as low as the last dorsal vertebra.

An examination disclosed great tenderness over the 5th cervical vertebra, but we could not discover any crepitus or inequality in the line of the spinous processes.

All of the symptoms remained the same until within a few minutes of his death, indeed his death occurred with no other premonition than a single prolonged inspiration.

I very much regret that we were unable to examine the part injured after death.

Yours truly,

F. H. Hamilton, M. D. C. M. Crandall.

Springville, Oct. 27, 1856.

*Dr. Hamilton:*

Allow me to address you in relation to an interesting case of dislocation of the femur into the foramen ovale—interesting to me from the part I was brought to act in it, and interesting to the profession on account of the attention which "Reid's process" has lately elicited.

The subject of the accident was a very strong muscular man, twenty-three years old, and weighing 175lbs. He was engaged in turning over a stump, his horses were hitched to it and he was standing under a pry lifting, while the horses were drawing and had nearly turned it, when, the chain slipping, it suddenly fell back upon the lever, striking him on the head, crushing him to the ground and dislocating the head of the right femur into the obturator muscle. The limb took the usual direction of rotation outward and elongation, with tension of the adductors, flatness of the trochanter, &c. When I arrived I found the family physician, Dr. Dresser, of the town of Otto, and six or eight men, who had been making transverse and longitudinal extension, but without accomplishing the reduction. I told them, before making farther extension, I would like to try *Reid's process.* Accordingly, placing him on his back, and having assistants to hold the pelvis firmly, I seized the right ankle with my right hand and the thigh with the left and flexed the thigh to within six inches of the body, then forcibly adducted the limb, but without moving the head of the bone. Then while I was bringing the thigh down to a right angle to the body, adducting the knee and abducting the heel, we felt it begin to move while I straightened the limb, and to slide, (as we supposed,) into the cotyloid cavity.

All pronounced it *set.* The limb when measured from the spinous process of the illium was as long as the other and was but slightly inverted, still it pained him, and he would not suffer it to be examined as much as desirable.

It being nearly sundown and having eight miles to ride before total darkness should supervene, I started home, all the way thinking over the case. The more I thought, the more fully I became convinced that I had only converted one form of dislocation into another. After a sleepless night, I hastened back to the scene of action, and found my fears fully realized. — The head of the femur was in the ischiatic notch! Having summoned his physician and other help we gave him chloroform, and I endeavored by reversing the process as in Dr. Mackoe's case, to bring the head back into the *foramen ovale*, but did not succeed.

Once after flexing the limb, (as you did in your case reported as successful,) in bringing it down, it met with the same kind of resistance, and produced a similar snapping sound and probably from a similar cause, but not having faith in a direct reduction, I did not persevere, as I should otherwise have done. I supposed the head must travel back the same road by which it had arrived in the notch. I then advised them to send for yourself or Dr. Colegrove. They concluded to call the latter. We accordingly met the next day and agreed to the following course, viz.: Tart. Ant. *ad nauseam* and bleeding from both arms in the erect posture *ad deliquium animi*, which was faithfully practiced. Then with sheet to the groin for counter extension, held firmly by two or three men, three to pull at the limb to extend, and myself to hold the pelvis and to make lateral extension, we soon accomplished a *direct* reduction as follows:

After sufficient extension had been made, the limb was flexed to near a right angle and the heel adducted so as to tilt up the head of the bone, while the lateral force being continued it leaped into its socket with a sensible jar, and the limb assumed its natural position.

I consider this reduction, under the direction of Dr. Colegrove, as a complete triumph of science. Here was a combination of the usual method with that of manipulation, without which the reduction, probably, would not have been effected. I am still of the opinion that such a dislocation might be reduced by manipulation alone, after producing nausea and faintness, or perhaps without, by being careful not to flex the limb to more than an angle of about 100 degrees with the body, then as in Dr. Mackoe's case in the N. Y. Hospital, first abduct the limb a little, in order to tilt up the head of the bone, so as to prevent its gliding below the acetabulum. Then carry the knee inward and abduct the heel, and the head would glide upward and outward to its home.

Truly your friend,

Dr. F. H. Hamilton. JOHN G. HOUSE.

